# Establishment and application of a prediction model for blastocyst formation rate and delivery outcome in human assisted reproductive technology

**DOI:** 10.1530/RAF-25-0021

**Published:** 2026-03-03

**Authors:** Meng-Dan Xi, Hong-Yi Xu, Xiao-Ning Wang, Jia-Rong Tian, Min Lu, Hong-Tao Zheng, Li He, Ying Zhang

**Affiliations:** ^1^ School of Basic Medicine, Hubei University of Medicine, Shiyan, China; ^2^ Reproductive Medicine Center, Renmin Hospital, Hubei University of Medicine, Shiyan, China; ^3^ Hubei Clinical Research Center for Reproductive Medicine, Shiyan, China; ^4^ Biomedical Engineering College, Hubei University of Medicine, Shiyan, China; ^5^ Hubei Key Laboratory of Embryonic Stem Cell Research, Hubei University of Medicine, Shiyan, China; ^6^ Biomedical Research Institute, Hubei University of Medicine, Shiyan, Hubei, China

**Keywords:** blastocyst formation rate, delivery outcome, assisted reproductive technology (ART), mathematical modeling

## Abstract

**Graphical Abstract:**

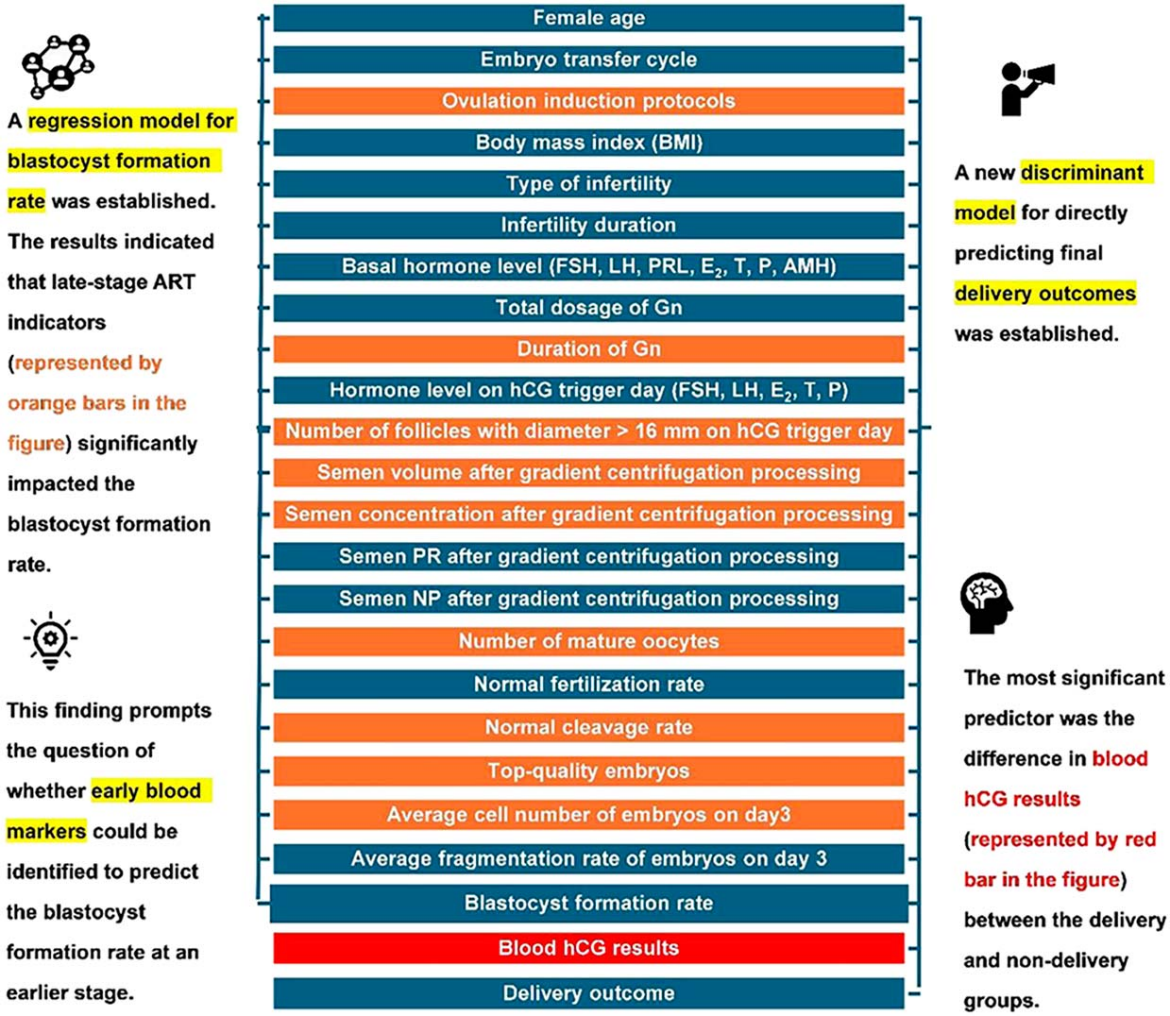

**Abstract:**

With the gradual emergence of population issues and the increasing incidence of infertility, the role of assisted reproductive technology (ART) is becoming increasingly important. Accurately, objectively, and comprehensively assessing the blastocyst formation rate and predicting delivery outcomes are urgent problems to be solved. This study retrospectively analyzed the clinical data of patients who underwent ART at Renmin Hospital, Hubei University of Medicine, from June 2017 to January 2020. A regression model for the blastocyst formation rate was established through regression analysis. The results showed that late-stage indicators in ART had a significant impact on the blastocyst formation rate, with top-quality embryos having the greatest effect. A discriminant analysis model for delivery outcome was established. It correctly classified 80.4% of the original grouped cases and 79.6% of the cross-validated grouped cases. The area under the receiver operating characteristic curve (AUROC) was 0.887, indicating that the discriminant model has a relatively high predictive diagnostic value. Analysis revealed that blood hCG (human chorionic gonadotropin) results play a crucial role in the discriminant analysis model. In addition, the number of top-quality embryos and the blastocyst formation rate also have a significant impact on the accurate prediction of delivery outcomes. Through analyzing the regression model, we propose exploring early blood markers that can predict the blastocyst formation rate. Simultaneously, a new discriminant model that can directly predict the final delivery outcomes was established.

**Lay summary:**

Against the backdrop of gradually emerging population issues and a rising incidence of infertility, assisted reproductive technology (ART) is playing an increasingly vital role. Accurately, objectively, and comprehensively evaluating embryo quality and predicting transplantation success rates represent pressing challenges that need to be addressed. This study retrospectively analyzed clinical data from patients who underwent ART. We developed a model that uses statistical methods to assess the formation of blastocysts (early-stage embryo). The results indicate that certain late-stage indicators in ART significantly influence the blastocyst formation rate. Therefore, we propose a new direction: exploring early blood markers that can predict blastocyst formation rate. Simultaneously, a new method was established to predict delivery outcomes, demonstrating strong predictive performance and high accuracy.

## Introduction

With the gradual emergence of population issues and the increasing incidence of infertility, the role of assisted reproductive technology is becoming increasingly important. Accurately, objectively, and comprehensively assessing the blastocyst formation rate and predicting delivery outcomes are currently urgent problems to be solved. The significant decline in fertility rates in various countries is due not only to social and economic factors but also to significant reproductive health issues (GBD 2021 Fertility and Forecasting Collaborators 2024). Various factors, such as air pollution, smoking, alcohol consumption, stress, and environmental hormones, can affect human fertility, causing great distress to many couples of childbearing age (Petrocnik *et al.* 2021). The emergence of ART has brought hope to many infertile families (Oliveira *et al.* 2021).

ART has made tremendous progress after more than 40 years of rapid development. However, its live birth rate remains suboptimal, and the pregnancy rate urgently needs improvement (Harper *et al.* 2018). According to the existing literature, multiple factors can affect the outcome of *in vitro* fertilization and pregnancy, including the woman’s age, body mass index (BMI), number of years of infertility, infertility factors, COH protocol, number of retrieved oocytes, number of transferable embryos, serum HCG concentration, and total dose of Gn (Chi *et al.* 2010, Gerber *et al.* 2020, Yang *et al.* 2021, Williams *et al.* 2022). Embryo quality directly affects the outcome of ART and is the key to its success (Siristatidis *et al.* 2018).

A number of studies have developed predictive models for *in vitro* fertilization (IVF) outcomes. Zhang *et al.* (2022) constructed an artificial neural network model (AUC = 0.794) and a LogitBoost model (AUC = 0.791) to predict the live-birth success rate of natural-cycle *in vitro* fertilization (NC-IVF). Yang *et al.* (2025) developed a multivariate logistic regression model (AUC = 0.75) to predict live birth using data from cases involving chromosomal abnormalities and recurrent miscarriage. Rajendran *et al.* (2025) developed FEMI (foundational IVF model for imaging), which achieved an area under the receiver operating characteristic curve (AUROC) > 0.75 for blastocyst ploidy prediction. Cimadomo *et al.* (2025) and Rajendran *et al.* (2024) each developed artificial intelligence-based models for predicting blastocyst development and ploidy. Du *et al.* (2024) established a long short-term memory (LSTM)-based morphokinetic model (AUC = 0.730) to predict blastocyst formation. While the models mentioned above have been empirically validated, their predictive performance – particularly as measured by the area under the curve (AUC) – still leaves room for improvement.

This study conducted data analysis based on routine detection indicators that may affect the outcome of *in vitro* fertilization and pregnancy in human assisted reproduction. It retrospectively analyzed the clinical data of patients who underwent ART at Shiyan People’s Hospital from June 2017 to January 2020. A regression prediction model for blastocyst formation rate and a discriminant model for birth outcome were established using regression analysis and discriminant analysis, respectively. The index parameters that significantly impact the blastocyst formation rate and birth outcome were analyzed to build a prediction model that can more efficiently and directly predict birth outcome, thereby providing a data reference for improving the transplantation success rate and pregnancy rate.

## Materials and methods

### Clinical data collection

**Figure 1:**
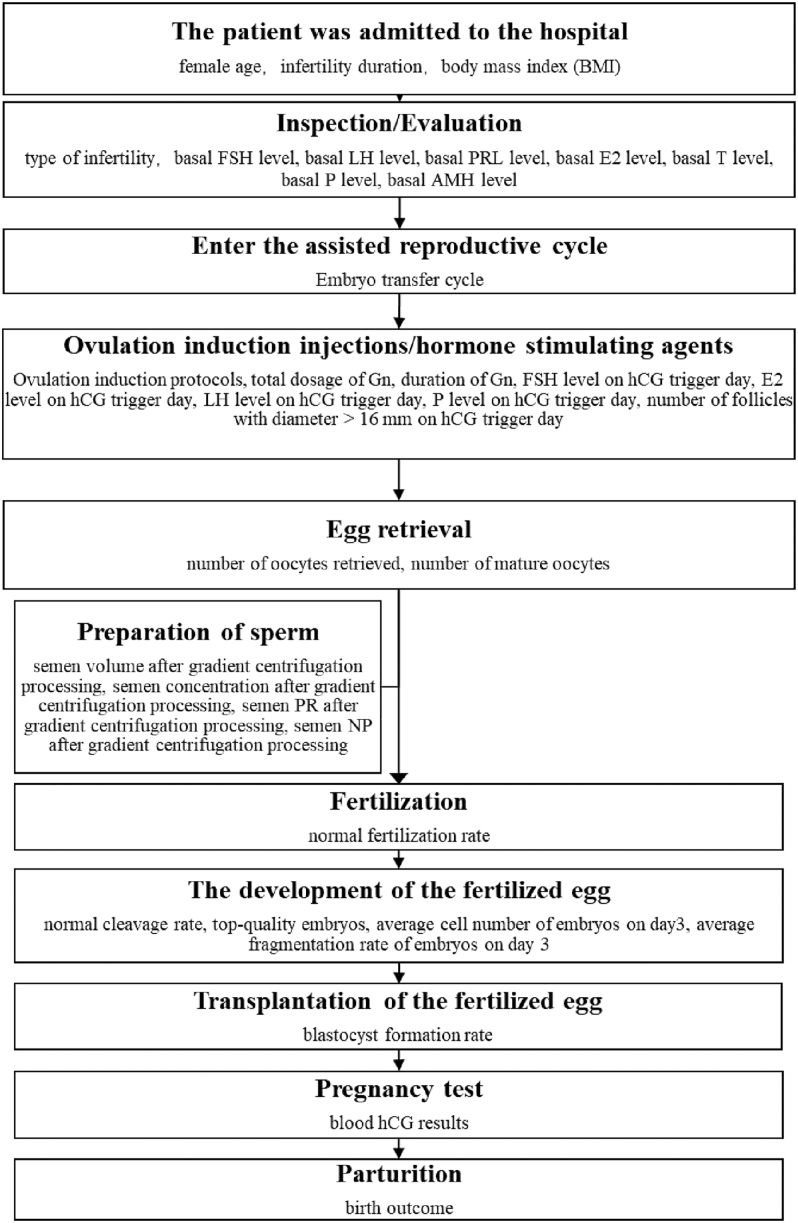
Flowchart of the indicator selection process.

The study population consisted of patients who visited the Reproductive Medicine Center, Renmin Hospital, Hubei University of Medicine, for ART. As shown in Fig. 1, after admission, basic patient information is recorded. Examinations are then conducted, and the results are recorded. Next, the assisted reproductive cycle is entered to record the number of embryo transfer cycles. Subsequently, controlled ovarian hyperstimulation (COH) is carried out to record the ovulation induction protocol and the use of stimulating hormones. Following another examination, oocytes are retrieved. Once basic sperm information is recorded, the sperm and oocytes are combined. The normal fertilization rate is observed and recorded. Embryo culture is uniformly performed using a cleavage medium. The development of fertilized oocytes and the blastocyst formation rate after culture are observed and recorded. Finally, pregnancy tests are conducted based on blood HCG test results, and the final delivery outcomes are recorded. The indicators recorded and incorporated into the model during this process include female age, embryo transfer cycle, ovulation induction protocols (categorical variable: 1 for COH0.1 long protocol and 2 for COH3.75 long protocol), BMI, type of infertility (categorical variable: 1 for primary infertility and 2 for secondary infertility), infertility duration, basal FSH (follicle-stimulating hormone) level, basal LH (luteotropic hormone) level, basal PRL (prolactin) level, basal E_2_ (estradiol) level, basal T (testosterone) level, basal P (progesterone) level, basal AMH (anti-Müllerian hormone) level, total dosage of Gn (gonadotropins), duration of Gn, FSH level on hCG trigger day, E_2_ level on hCG trigger day, LH level on hCG trigger day, progesterone level on hCG trigger day, number of follicles with diameter > 16 mm on hCG trigger day, semen volume after gradient centrifugation processing, semen concentration after gradient centrifugation processing, semen PR (progressive motility) after gradient centrifugation processing, semen NP (non-progressive motility) after gradient centrifugation processing, number of mature oocytes, normal fertilization rate, normal cleavage rate, top-quality embryos (defined as embryos evaluated and screened during IVF, prioritized as those with 8 cells on day 3, uniform blastomeres, single nucleus, few fragments, and normal development speed), average cell number of embryos on day 3, average fragmentation rate of embryos on day 3, blastocyst formation rate (continuous variable), blood HCG results, and delivery outcome (categorical variable: 1 for no delivery and 2 for successful delivery of live fetus). The first 31 indicators are included in the linear regression model, while all 33 indicators are included in the discriminant model. Of these 33 indicators, except for ovulation induction protocol, type of infertility, and delivery outcome, which are categorical variables, the other 30 are continuous variables.

### Ethics approval

We confirm that all methods were carried out in accordance with relevant guidelines and regulations, and all experimental protocols were approved by the Reproductive Medicine Ethics Committee of Shiyan People’s Hospital.

### Clinical data analysis and modeling

Clinical data from patients undergoing ART in our center from June 2017 to January 2020 were retrospectively analyzed. A total of 3,430 patients with complete and accurate records of the above indicators were selected. Timely recording by designated personnel after data detection was adopted to avoid misrecording. Records with missing indicators were directly excluded. The clinical data were analyzed, and a linear regression model for blastocyst formation rate was established. The effects of each index in the regression model on the blastocyst formation rate were analyzed.

Subsequently, a discriminant model for childbirth outcome was established. The index parameters that significantly impacted the childbirth outcome were analyzed. The sample size for discriminant analysis was 2,779 patients.

### Statistical analysis

Data were analyzed using SPSS 16.0 for Windows. The linear regression model for blastocyst formation rate was established by regression analysis. The discriminant model for delivery outcome was established by discriminant analysis.

### Model parameter analysis

The reliability and prediction efficiency of the blastocyst formation rate regression model were analyzed using model parameters. i) The variance inflation factor (VIF) is greater than 1. The closer the VIF value is to 1, the weaker the multicollinearity. The farther the VIF value is from 1, the stronger the multicollinearity. Severe multicollinearity makes significance tests of variables meaningless and reduces the stability of regression models. Therefore, the VIF value should be as close to 1 as possible. ii) The Durbin–Watson statistic is used to determine the independence of the model data. The value is generally around 2, ideally between 1.5 and 2.5. Therefore, the Durbin–Watson value should be as close to 2 as possible. iii) The adjusted *R^2^* of the model represents the proportion of variance in the dependent variable explained by the independent variables. Generally, a model is considered acceptable when the adjusted *R*^*2*^ is > 0.3.

The discriminant model built for live birth prediction uses the area under the receiver operating characteristic curve (AUROC) for model performance assessment (e.g. sensitivity and specificity).

## Results

All indicators for the participants in this study were recorded in detail without any missing items. The female participants were aged 20–43 years, the number of embryo transfer cycles ranged from 1 to 7, BMI ranged from 14.587 to 42.188, the number of mature oocytes ranged from 2 to 21, the normal fertilization rate ranged from 33.33 to 100%, the normal cleavage rate ranged from 50 to 100%, and the number of top-quality embryos ranged from 0 to 10. The average cell number of embryos on day 3 ranged from 2.5 to 11, the average fragmentation rate of embryos on day 3 ranged from 5 to 74%, the blastocyst formation rate ranged from 0 to 100%, and the blood HCG result ranged from 0 to 12,578.6.

### Establishment of regression model for predicting blastocyst formation rate

The collected clinical data were analyzed using SPSS multiple linear regression analysis. The obtained regression model was as follows: blastocyst formation rate = −87.842–0.109* female age −2.534* embryo transfer cycle +4.391* ovulation induction protocols +0.02* BMI +0.431* type of infertility +0.148* infertility duration +0.325* basal FSH level −0.018* basal LH level +0.011* basal PRL level +0.001* basal E_2_ level +0.187* basal testosterone level-0.019* basal progesterone level-0.046* basal AMH level +0.001* total dosage of Gn −1.377* duration of Gn −0.023* FSH level on hCG trigger day −0.00014* E_2_ level on hCG trigger day −0.012* LH level on hCG trigger day +0.008* Progesterone level on hCG trigger day −0.428* number of follicles with diameter > 16 mm on hCG trigger day −7.178* semen volume after gradient centrifugation processing +0.098* semen concentration after gradient centrifugation processing −0.016* semen PR after gradient centrifugation processing −0.242* semen NP after gradient centrifugation processing +3.196* number of mature oocytes +0.029* normal fertilization rate +0.742* normal cleavage rate +6.961* top-quality embryos +6.494* average cell number of embryos on day 3 −0.018* average fragmentation rate of embryos on day 3. The parameters of the regression model were as follows: most VIF values were close to 1, the Durbin–Watson value was 1.952, and the adjusted *R*^*2*^ was 0.328, indicating good reliability and prediction efficiency of the model.

The significance level (*P* value) of each index in the linear regression model for blastocyst formation rate is shown in Table 1.

**Table 1:** Coefficient α of blastocyst formation rate regression model. Statistically significant differences (*P < *0.05) are presented in bold.

Model	Unstandardized coefficients		*t*	*P* value	Collinearity statistics	
	B	SE			TOL	VIF
Constant	−87.842	16.901	−5.197	**2.14 × 10^−7^**		
Female age	−0.109	0.168	−0.649	0.516	0.772	1.296
Embryo transfer cycle	−2.534	1.346	−1.883	0.060	0.972	1.028
Ovulation induction protocols	4.391	1.590	2.762	**0.006**	0.673	1.486
BMI	0.02	0.163	0.122	0.903	0.706	1.416
Type of infertility	0.431	1.171	0.368	0.713	0.869	1.150
Infertility duration	0.148	0.236	0.626	0.531	0.879	1.138
Basal FSH level	0.325	0.282	1.152	0.250	0.666	1.503
Basal LH level	−0.018	0.070	−0.255	0.799	0.630	1.587
Basal PRL level	0.011	0.020	0.541	0.589	0.992	1.008
Basal E_2_ level	0.001	0.008	0.171	0.864	0.736	1.358
Basal testosterone level	0.187	0.123	1.520	0.129	0.986	1.014
Basal progesterone level	−0.019	0.041	−0.450	0.652	0.961	1.041
Basal AMH level	−0.046	0.043	−1.074	0.283	0.961	1.041
Total dosage of Gn	0.001	0.001	1.326	0.185	0.339	2.946
Duration of Gn	−1.377	0.469	−2.936	**0.003**	0.409	2.444
On hCG trigger day						
FSH level	−0.023	0.013	−1.688	0.092	0.978	1.022
E_2_ level	−0.00014	0.00033	−0.437	0.662	0.775	1.290
LH level	−0.012	0.013	−0.917	0.359	0.993	1.007
Progesterone level	0.008	0.047	0.165	0.869	0.993	1.007
Number of follicles with diameter > 16 mm	−0.428	0.183	−2.342	**0.019**	0.778	1.285
After gradient centrifugation processing						
Semen volume	−7.178	2.697	−2.661	**0.008**	0.946	1.057
Semen concentration	0.098	0.044	2.228	**0.026**	0.492	2.034
Semen PR	−0.016	0.031	−0.501	0.616	0.426	2.345
Semen NP	−0.242	0.204	−1.186	0.236	0.713	1.403
Number of mature oocytes	3.196	0.263	12.167	**2.29 × 10^−33^**	0.483	2.069
Normal fertilization rate	0.029	0.046	0.630	0.529	0.908	1.101
Normal cleavage rate	0.742	0.132	5.637	**1.87 × 10^−8^**	0.951	1.051
Top-quality embryos	6.961	0.457	15.245	**8.27 × 10^−51^**	0.459	2.180
Average number of embryo cells on day 3	6.494	0.696	9.325	**1.95 × 10^−20^**	0.685	1.460
Average fragmentation rate of embryos on day 3	−0.018	0.074	−0.248	0.804	0.777	1.287

SE, standard error; TOL, tolerance; and BMI, body mass index.

The analysis results of the model parameters are as follows: i) most VIF values are close to 1 and no VIF value exceeded 3. This indicates no multicollinearity among the indexes in the model, and the model has high reliability. ii) The Durbin–Watson value was 1.952, very close to 2, indicating strong independence of the model data. iii) The adjusted *R*^*2*^ of the model was 0.328 > 0.3, indicating that the independent variables have good explanatory power for the dependent variable (blastocyst formation rate).

Based on the data in Table 1 and the linear regression equation, the specific interpretation of the relationship between each independent variable and the blastocyst formation rate is as follows. Assuming that the other independent variables remain constant, the following conclusions can be drawn regarding a change in a single independent variable: for every additional year of female age, the blastocyst formation rate decreases by an average of 0.109 percentage points. For each additional embryo transfer cycle, the blastocyst formation rate decreases by an average of 2.534 percentage points. The average blastocyst formation rate of the COH3.75 ovulation induction protocol is 4.391 percentage points higher than that for the COH0.1 protocol. For every one-unit increase in BMI, the blastocyst formation rate increases by an average of 0.02 percentage points. On average, the blastocyst formation rate for secondary infertility is 0.431 percentage points higher than that for primary infertility. For each additional year of infertility duration, the blastocyst formation rate increases by an average of 0.148 percentage points. For every one-unit increase in basal FSH level, the blastocyst formation rate increases by an average of 0.325 percentage points. For every one-unit increase in basal LH level, the blastocyst formation rate decreases by an average of 0.018 percentage points. For every one-unit increase in basal PRL level, the blastocyst formation rate increases by an average of 0.011 percentage points. For every one-unit increase in basal E_2_ level, the blastocyst formation rate increases by an average of 0.001 percentage points. For every one-unit increase in basal testosterone level, the blastocyst formation rate increases by an average of 0.187 percentage points. For every one-unit increase in basal progesterone level, the blastocyst formation rate decreases by an average of 0.019 percentage points. For every one-unit increase in basal AMH level, the blastocyst formation rate decreases by an average of 0.046 percentage points. For every one-unit increase in the total dosage of Gn, the blastocyst formation rate increases by an average of 0.001 percentage points. For each additional day of Gn duration, the blastocyst formation rate decreases by an average of 1.377 percentage points. For every one-unit increase in FSH level on hCG trigger day, the blastocyst formation rate decreases by an average of 0.023 percentage points. For every one-unit increase in E_2_ level on hCG trigger day, the blastocyst formation rate decreases by an average of 0.00014 percentage points. For every one-unit increase in LH level on hCG trigger day, the blastocyst formation rate decreases by an average of 0.012 percentage points. For every one-unit increase in progesterone level on hCG trigger day, the blastocyst formation rate increases by an average of 0.008 percentage points. For every one-unit increase in the number of follicles with diameter > 16 mm on hCG trigger day, the average blastocyst formation rate decreases by 0.428 percentage points. For every unit increase in semen volume after gradient centrifugation processing, the blastocyst formation rate decreases by an average of 7.178 percentage points. For every one-unit increase in semen concentration after gradient centrifugation processing, the blastocyst formation rate increases by an average of 0.098 percentage points. For every additional unit of semen PR after gradient centrifugation processing, the blastocyst formation rate decreases by an average of 0.016 percentage points. For every additional unit of semen NP after gradient centrifugation processing, the blastocyst formation rate decreases by an average of 0.242 percentage points. For every one-unit increase in the number of mature oocytes, the blastocyst formation rate increases by an average of 3.196 percentage points. For every one-unit increase in the normal fertilization rate, the blastocyst formation rate increases by an average of 0.029 percentage points. For every one-unit increase in the normal cleavage rate, the blastocyst formation rate increases by an average of 0.742 percentage points. For each additional top-quality embryo, the blastocyst formation rate increases by an average of 6.961 percentage points. For every one-unit increase in the average cell number of embryos on day 3, the blastocyst formation rate increases by an average of 6.494 percentage points. For every one unit increase in the average fragmentation rate of embryos on day 3, the blastocyst formation rate decreases by an average of 0.018 percentage points.

Meanwhile, based on the data results, we found that i) the indexes that had a significant influence on the blastocyst formation rate were ovulation induction protocols, duration of Gn, number of follicles with diameter > 16 mm on hCG trigger day, semen volume after gradient centrifugation processing, semen concentration after gradient centrifugation processing, number of mature oocytes, normal cleavage rate, top-quality embryos, and average cell number of embryos on day 3. ii) The late detection indexes in ART were the main indicators that had a very significant effect on the blastocyst formation rate. iii) Top-quality embryos had the greatest effect on the blastocyst formation rate in this regression model, exhibiting a very significant positive effect.

Although the regression model has good predictive ability, we found that its performance could be further improved by removing several indicators with a very low influence on the blastocyst formation rate. Therefore, we eliminated 21 indicators using the ‘stepwise method’ in linear regression analysis, including female age, embryo transfer cycle, BMI, type of infertility, infertility duration, basal FSH level, basal LH level, basal PRL level, basal E_2_ level, basal testosterone level, basal progesterone level, basal AMH level, total dosage of Gn, FSH level on hCG trigger day, E_2_ level on hCG trigger day, LH level on hCG trigger day, progesterone level on hCG trigger day, semen PR after gradient centrifugation processing, semen NP after gradient centrifugation processing, normal fertilization rate, and average fragmentation rate of embryos on day 3. Using the nine remaining indicators as independent variables, a new regression model for blastocyst formation rate was obtained: blastocyst formation rate = −93.454 + 4.083* ovulation induction protocols −0.841* duration of Gn −0.466* number of follicles with diameter > 16 mm on hCG trigger day − 7.347* semen volume after gradient centrifugation processing +0.102* semen concentration after gradient centrifugation processing +3.082* number of mature oocytes +0.746* normal cleavage rate +7.034* top-quality embryos +6.537* average cell number of embryos on day 3. The adjusted *R*^*2*^ of the new regression model is 0.329, slightly higher than that of the model before removal, while other parameters remained largely unchanged, indicating that removing low-impact indicators did not significantly improve model quality.

### Establishment of discriminant model for predicting delivery outcome

Clinical data from ART treatments were collected and collated. The delivery outcome was a categorical variable: 1 for no delivery; 2 for successful delivery of a live fetus. SPSS software was used for discriminant analysis.

In the output results, the equality test of group means was first analyzed (Table 2). The results showed that the most significant differences between the two groups were in female age, ovulation induction protocols, infertility duration, FSH level on hCG trigger day, number of mature oocytes, normal fertilization rate, top-quality embryos, average cell number of embryos on day 3, blastocyst formation rate, and blood hCG results. There were significant differences in embryo transfer cycle, BMI, basal PRL level, basal E_2_ level, basal AMH level, total dosage of Gn, and E_2_ level on hCG trigger day between the delivery and non-delivery groups. The differences in top-quality embryos, blastocyst formation rate, and blood hCG results between the delivery and non-delivery groups were extremely significant.

**Table 2:** Tests of equality of group means for determining childbirth outcomes. Statistically significant differences (*P < *0.05) are presented in bold.

	Wilks’ lambda	F	*P* value
Female age	0.994	17.856	**2.459 × 10^−5^**
Embryo transfer cycle	1.000	0.228	0.633
Ovulation induction protocols	0.993	18.425	**1.828 × 10^−5^**
BMI	0.998	5.548	**0.019**
Type of infertility	1.000	5.552	0.458
Infertility duration	0.997	8.103	**0.004**
Basal FSH level	0.999	1.449	0.229
Basal LH level	1.000	0.558	0.455
Basal PRL level	0.999	3.925	**0.048**
Basal E_2_ level	0.998	4.804	**0.028**
Basal testosterone level	1.000	0.00023	0.988
Basal progestone level	1.000	0.390	0.532
Basal AMH level	0.998	4.469	**0.035**
Total dosage of Gn	0.999	3.915	**0.048**
Duration of Gn	1.000	0.102	0.749
On hCG trigger day			
FSH level	0.994	16.903	**4.047 × 10^−5^**
E_2_ level	0.998	6.287	**0.012**
LH level	1.000	0.115	0.734
Progesterone level	1.000	0.654	0.419
Number of follicles with diameter > 16 mm	1.000	0.582	0.446
After gradient centrifugation processing			
Semen volume	0.999	2.765	0.096
Semen concentration	1.000	0.241	0.623
Semen PR	1.000	0.736	0.391
Semen NP	1.000	0.082	0.775
Number of mature oocytes	0.984	44.909	**2.492 × 10^−11^**
Normal fertilization rate	0.996	9.979	**0.002**
Normal cleavage rate	1.000	0.726	0.394
Top-quality embryos	0.969	87.267	**1.888 × 10^−20^**
Average number of embryo cellss on day 3	0.989	31.334	**2.385 × 10^−8^**
Average fragmentation rate of embryos on day 3	1.000	0.268	0.604
Blastocyst formation rate	0.965	99.994	**3.757 × 10^−23^**
Blood hCG results	0.723	1,063.351	**1.115 × 10^−197^**

BMI, body mass index.

Two methods are provided in the discriminant analysis output to predict the delivery outcome: the first is to write the classification function for each group; the case is assigned to the group for which the calculated value is larger.

Non-delivery group = −484.251 + 2.386* female age +10.286* embryo transfer cycle +1.503* ovulation induction protocols +2.915* BMI +2.871* type of infertility −0.07* infertility duration +2.084* basal FSH level −0.202* basal LH level −0.007* basal PRL level +0.056* basal E_2_ level +0.163* basal testosteone level +0.073* basal progesterone level −0.017* basal AMH level −0.015* total dosage of Gn +6.706* duration of Gn +0.505* FSH level on hCG trigger day +0.0004758* E_2_ level on hCG trigger day +0.183* LH level on hCG trigger day −0.049* progesterone level on hCG trigger day +1.015* number of follicles with diameter > 16 mm on hCG trigger day +5.112* semen volume after gradient centrifugation processing +0.145* semen concentration after gradient centrifugation processing +0.205* semen PR after gradient centrifugation processing +1.351* semen NP after gradient centrifugation processing +2.089* number of mature oocytes +0.79* normal fertilization rate +5.702* normal cleavage rate −5.331* top-quality embryos +10.217* average cell number of embryos on day 3 +0.226* average fragmentation rate of embryos on day 3 −0.077* blastocyst formation rate +0.00002799* blood hCG results.

Delivery group = −485.93 + 2.364* female age +10.304* embryo transfer cycle +1.709* ovulation induction protocols +2.961* BMI +2.953* type of infertility −0.076* infertility duration +2.105* basal FSH level −0.1998* basal LH level −0.011* basal PRL level +0.055* basal E_2_ level +0.155* basal testosterone level +0.077* basal progesterone level −0.014* basal AMH level −0.015* total dosage of Gn +6.72* duration of Gn +0.501* FSH level on hCG trigger day +0.000425* E_2_ level on hCG trigger day +0.195* LH level on hCG trigger day −0.048* progesterone level on hCG trigger day +0.992* number of follicles with diameter > 16 mm on hCG trigger day +4.838* semen volume after gradient centrifugation processing +0.146* semen concentration after gradient centrifugation processing +0.203* semen PR after gradient centrifugation processing +1.36* semen NP after gradient centrifugation processing +2.106* number of mature oocytes +0.799* normal fertilization rate +5.699* normal cleavage rate −5.345* top-quality embryos +10.189* average cell number of embryos on day 3 +0.22* average fragmentation rate of embryos on day 3 −0.074* blastocyst formation rate +0.00111* blood hCG results.

The second method is to use the typical discriminant function provided in the output: discriminant score = −1.461–0.017* female age +0.014* embryo transfer cycle +0.159* ovulation induction protocols +0.036* BMI +0.063* type of infertility −0.005* infertility duration +0.016* basal FSH level +0.002* basal LH level −0.003* basal PRL level −0.001* basal E_2_ level −0.006* basal testosterone level +0.004* basal progesterone level +0.003* basal AMH level −0.00009* total dosage of Gn +0.011* duration of Gn −0.003* FSH level on hCG trigger day −0.0000389* E_2_ level on hCG trigger day +0.01* LH level on hCG trigger day +0.001* progesterone level on hCG trigger day −0.017* number of follicles with diameter > 16 mm on hCG trigger day −0.211* semen volume after gradient centrifugation processing +0.001* semen concentration after gradient centrifugation processing −0.001* semen PR after gradient centrifugation processing +0.007* semen NP after gradient centrifugation processing +0.013* number of mature oocytes +0.006* normal fertilization rate −0.002* normal cleavage rate −0.011* top-quality embryos −0.022* average cell number of embryos on day 3 −0.005* average fragmentation rate of embryos on day 3 +0.003* blastocyst formation rate +0.001* blood hCG results.

Next, the group centroids (−0.696 for the non-delivery group and 0.599 for the delivery group) are subtracted from the calculated discriminant score for each case. The absolute value of the difference is taken, and the case is assigned to the group with the smallest absolute difference.

**Figure 2:**
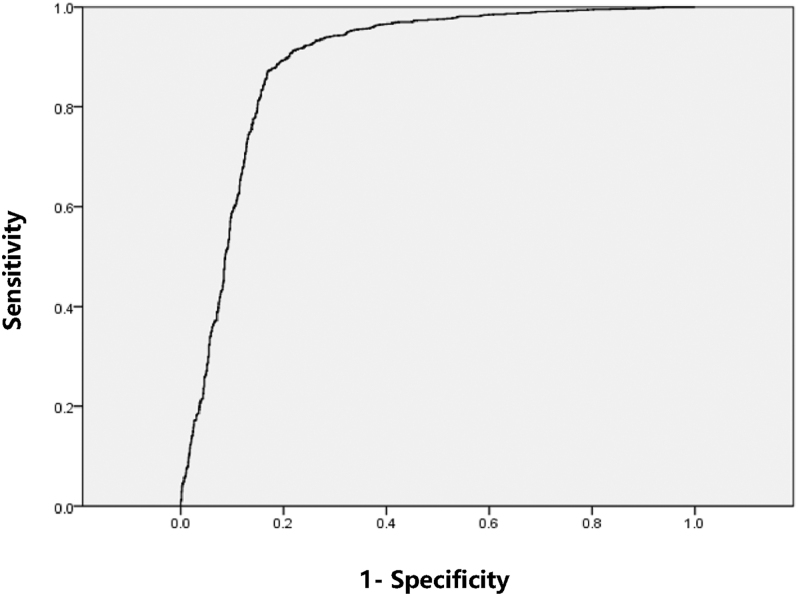
ROC curve.

Through data analysis of the discriminant model, 80.4% of original grouped cases were correctly classified and 79.6% of cross-validated grouped cases were correctly classified. Cross-validation is performed only for the cases in the analysis. In cross-validation, each case is classified by the functions derived from all other cases. The discriminant model for live birth prediction was assessed using the AUROC. The ROC curve (Fig. 2) was used to evaluate the discriminant model, yielding an area under the curve of 0.887 (AUC = 0.887; 95% CI = 0.874–0.901; *P* = 3.26 × 10^−271^ < 0.01; the maximum Youden index was 0.701896067783611, at which point the diagnostic test had the best sensitivity of 0.87038280725319 and specificity of 0.831513260530421). These results indicate that the predictive diagnostic value and discriminative accuracy of the model are high. When blood HCG results were excluded from the discriminant model, the proportion of correctly classified cases in the original group decreased from 80.4 to 61.6%, and the cross-validated accuracy decreased from 79.6 to 60.3%; the area under the curve also decreased from 0.887 to 0.646 (AUC = 0.646; 95% CI = 0.626–0.667; *P* = 2.68 × 10^−40^ < 0.01).

## Discussion

The indicators that significantly influenced the blastocyst formation rate were ovulation induction protocols, duration of Gn, number of follicles with diameter > 16 mm on hCG trigger day, semen volume after gradient centrifugation processing, semen concentration after gradient centrifugation processing, number of mature oocytes, normal cleavage rate, top-quality embryos, and average cell number of embryos on day 3.

Compared with female age, BMI, embryo transfer cycle, infertility duration, and type of infertility, the ovulation induction protocols had a more significant effect on the blastocyst formation rate (*P* = 0.006). The blastocyst formation rate of the COH3.75 ovulation induction protocol was extremely significantly higher than that of the COH0.1 protocol.

The results showed a negative correlation between the duration of Gn use and the blastocyst formation rate (*P* = 0.003); the coefficient for Gn duration in the regression model was −1.377. The relationship between Gn duration and blastocyst formation rate has not been definitively established. Some scholars have reviewed the correlation between the duration of gonadotropin use and IVF outcome. According to the literature review (Mu *et al.* 2019), prolonged use of gonadotropin-releasing hormone agonist (GnRH-a) could excessively inhibit LH and decrease ovarian responsiveness, oocyte quality, and embryo quality, supporting the concept of an LH ‘threshold’ proposed by Balasch & Fábregues (2002). These findings have substantial implications for optimizing clinical ovulation induction protocols.

Neither the basal hormone levels in serum nor the hormone levels on the hCG trigger day were significantly correlated with the blastocyst formation rate. The number of follicles with diameter > 16 mm on the hCG trigger day showed a negative correlation with the blastocyst formation rate (*P* = 0.019). The reasons for this phenomenon remain to be analyzed. A possible reason is that, due to the influence of progesterone and other factors, there might be a spurious correlation between the FSH level on the hCG trigger day and the blastocyst formation rate. According to the literature review, follicles with a diameter of ≥ 14 mm on the hCG trigger day are involved in progesterone secretion. An increase in progesterone secretion by follicles can result from the interruption of follicular development or maturation, thereby reducing the likelihood of pregnancy. A high progesterone-to-follicle index (PFI) adversely affects IVF implantation and pregnancy outcomes (Shufaro *et al.* 2015).

Of the various semen detection indexes, semen volume (*P* = 0.008) and concentration (*P* = 0.026) after gradient centrifugation processing had significant effects on the blastocyst formation rate. However, semen PR and NP after gradient centrifugation processing, which reflect sperm motility, did not significantly affect the blastocyst formation rate. This suggests that semen volume has a greater influence on the blastocyst formation rate than sperm motility in ART.

The indicators that have a very significant impact on the blastocyst formation rate are mostly concentrated in the late detection indicators in ART (Fig. 1), such as number of mature oocytes (*P* = 2.29 × 10^−33^), normal cleavage rate (*P* = 1.87 × 10^−8^), top-quality embryos (*P* = 8.27 × 10^−51^), and average cell number of embryos on day 3 (*P* = 1.95 × 10^−20^). Of these, top-quality embryos have the greatest influence, exhibiting a very significant positive effect. The higher the proportion of top-quality embryos, the higher the blastocyst formation rate. However, in clinical practice, early indicators have more predictive value and guiding significance than late indicators. Finding effective early indicators would be more relevant. Therefore, future studies should actively explore and screen for early blood markers that can accurately predict embryo quality.

Through data analysis of the discriminant model, 80.4% of original grouped cases were correctly classified and 79.6% of cross-validated grouped cases were correctly classified; the AUROC was 0.887. In the results of the discriminant model (Table 2), significant differences between the delivery and non-delivery groups were observed for female age (*P* = 2.459 × 10^−5^), ovulation induction protocols (*P* = 1.828 × 10^−5^), BMI (*P* = 0.019), infertility duration (*P* = 0.004), FSH level on hCG trigger day (*P* = 4.047 × 10^−5^), number of mature oocytes (*P* = 2.492 × 10^−11^), normal fertilization rate (*P* = 0.002), top-quality embryos (*P* = 1.888 × 10^−20^), average number of embryo cells on day 3 (*P* = 2.385 × 10^−8^), blastocyst formation rate (*P* = 3.757 × 10^−23^), blood hCG results (*P* = 1.115 × 10^−197^), basal PRL level (*P* = 0.048), basal E_2_ level (*P* = 0.028), basal AMH level (*P* = 0.035), total dosage of Gn (*P* = 0.048), and E_2_ level on hCG trigger day (*P* = 0.012). Of these, the difference in blood HCG results was the most significant. After excluding blood HCG results from the discriminant model, the proportion of correctly classified cases in the original group decreased from 80.4 to 61.6%, and the area under the curve decreased from 0.887 to 0.646. This indicates that blood HCG results play a crucial role in the accuracy of the discriminant model for predicting delivery outcome. In clinical practice, blood hCG results are a standard diagnostic tool for detecting pregnancy (Greene & Grenache 2015).

Through regression analysis and discriminant analysis (Tables 1 and 2), it was found that ovulation induction protocols, number of mature oocytes, top-quality embryos, and average cell number of embryos on day 3 not only significantly influenced the blastocyst formation rate but also presented significant differences between the delivery and non-delivery groups. Of these, the number of top-quality embryos was the most prominent. It not only had a significantly positive effect on the blastocyst formation rate but also exhibited a significant difference between the outcome groups, exerting a considerable impact on the precise identification of delivery results. However, the prevailing method for embryo selection remains traditional morphological scoring. While diverse morphological evaluation methods exist, traditional scoring is constrained by subjective factors, lacks dynamic and continuous observation, and has limited predictive accuracy. Time-lapse imaging addressed the issue of dynamic continuity, but its safety has not been definitively established. In recent years, emerging artificial intelligence (AI) technology can overcome the limitations of dynamic continuity and prediction accuracy; however, due to variations in indicator data and operating procedures among reproductive centers, the applicability of AI systems is not high (Si *et al.* 2023). Therefore, future research should actively explore employing AI technology, particularly deep learning, to establish more robust embryo selection models.

## Declaration of interest

The authors declare that there is no conflict of interest that could be perceived as prejudicing the impartiality of the research reported.

## Funding

This work was supported by the Natural Science Foundation of Hubei Province, Shiyan Innovation and Development Joint Fund Project (grant number 2024AFD114, JCZRLH202400751).

## Author contribution statement

XMD conceived the study, conducted the data analysis, and wrote the manuscript. XHY performed experiments, collected data, and was responsible for communication. WXN performed experiments. TJR performed experiments. LM assisted with the application of related projects. ZHT provided suggestions for the application of related projects. HL provided suggestions for the application of related projects. ZY provided the experimental platform, performed experiments, and promoted the research process.

## Data availability

The datasets used and/or analyzed during the current study are available from the corresponding author upon reasonable request.

## Informed consent

The authors confirm that informed consent was obtained from all subjects and/or their legal guardian(s).

## References

[bib1] Balasch J & Fábregues F 2002 Is luteinizing hormone needed for optimal ovulation induction? Curr Opin Obstet Gynecol 14 265–274. (10.1097/00001703-200206000-00004) 12032381

[bib2] Cimadomo D, Badajoz V, Hebles M, et al. 2025 Artificial intelligence-based donor oocyte quality assessment moderately improves the prediction of blastocyst development: a first step towards higher personalization in the management of egg donation treatments. Hum Reprod 40 1886–1892. (10.1093/humrep/deaf153) 40759152

[bib3] Chi H, Qiao J, Li H, et al. 2010 Double measurements of serum HCG concentration and its ratio may predict IVF outcome. Reprod Biomed Online 20 504–509. (10.1016/j.rbmo.2010.01.005) 20207583

[bib4] Du Y, Wang R, Liu Y, et al. 2024 Prediction of blastocyst formation based on fusion of morphokinetic and morphological features. J Appl Phys 136 12. (10.1063/5.0226639)

[bib5] GBD 2021 Fertility and Forecasting Collaborators 2024 Global fertility in 204 countries and territories, 1950–2021, with forecasts to 2100: a comprehensive demographic analysis for the global burden of disease study 2021. Lancet 403 2057–2099. (10.1016/S0140-6736(24)00550-6) 38521087 PMC11122687

[bib6] Gerber RS, Fazzari M, Kappy M, et al. 2020 Differential impact of controlled ovarian hyperstimulation on live birth rate in fresh versus frozen embryo transfer cycles: a society for assisted reproductive technology clinic outcome system study. Fertil Steril 114 1225–1231. (10.1016/j.fertnstert.2020.06.021) 33012553

[bib7] Greene DN & Grenache DG 2015 Pathology consultation on human chorionic gonadotropin testing for pregnancy assessment. Am J Clin Pathol 144 830–836. (10.1309/ajcp7o7vareduyij) 26572988

[bib8] Harper J, Aittomä ki K, Borry P, et al. 2018 Recent developments in genetics and medically assisted reproduction: from research to clinical applications. Eur J Hum Genet 26 12–33. (10.1038/s41431-017-0016-z) 29199274 PMC5839000

[bib9] Mu ZN, Sun ZG, Song JY, et al. 2019 Effect of duration of gonadotropin releasing hormone agonist on the outcome of in vitro fertilization-embryo transfer in a short-acting long regimen. Libyan J Med 14 1652058. (10.1080/19932820.2019.1652058) 31405338 PMC8896834

[bib10] Oliveira BL, Ataman LM, Rodrigues JK, et al. 2021 Restricted access to assisted reproductive technology and fertility preservation: legal and ethicalissues. Reprod Biomed Online 43 571–576. (10.1016/j.rbmo.2021.06.018) 34332903

[bib11] Petrocnik P, Mivsek AP, Zvanut B, et al. 2021 Preconception health in current society: the PreconNet project. Eur J Midwifery 5 6. (10.18332/ejm/132714) 33659868 PMC7910810

[bib12] Rajendran S, Brendel M, Barnes J, et al. 2024 Automatic ploidy prediction and quality assessment of human blastocysts using time-lapse imaging. Nat Commun 15 7756. (10.1038/s41467-024-51823-7) 39237547 PMC11377764

[bib13] Rajendran S, Rehani E, Phu W, et al. 2025 A foundational model for in vitro fertilization trained on 18 million time-lapse images. Nat Commun 16 6235. (10.1038/s41467-025-61116-2) 40645954 PMC12254344

[bib14] Si KY, Huang B & Jin L 2023 Application of artificial intelligence in gametes and embryos selection. Hum Fertil 26 757–777. (10.1080/14647273.2023.2256980) 37705466

[bib15] Shufaro Y, Sapir O, Oron G, et al. 2015 Progesterone-to-follicle index is better correlated with in vitro fertilization cycle outcome than blood progesterone level. Fertil Steril 103 669–674. (10.1016/j.fertnstert.2014.11.026) 25544249

[bib16] Siristatidis C, Sertedaki E, Vaidakis D, et al. 2018 Metabolomics for improving pregnancy outcomes in women undergoing assisted reproductive technologies. Cochrane Database Syst Rev 3 CD011872. (10.1002/14651858.CD011872.pub2) 28534597 PMC6481756

[bib17] Williams RS, Ellis DD, Wilkinson EA, et al. 2022 Factors affecting live birth rates in donor oocytes from commercial egg banks vs. program egg donors: an analysis of 40,485 cycles from the society for assisted reproductive technology registry in 2016–2018. Fertil Steril 117 339–348. (10.1016/j.fertnstert.2021.10.006) 34802685

[bib18] Yang R, Niu ZR, Chen LX, et al. 2021 Analysis of related factors affecting cumulative live birth rates of the first ovarian hyperstimulation in vitro fertilization or intracytoplasmic sperm injection cycle: a population-based study from 17978 women in China. Chin Med J 134 1405–1415. (10.1097/cm9.0000000000001586) 34091521 PMC8213303

[bib19] Yang Y, Zhang X, Zhang Y, et al. 2025 Chromosomal miscarriage and pregnancy outcomes in recurrent pregnancy loss. Reprod Fertil 6 e250052. (10.1530/raf-25-0052) 41283765 PMC12694013

[bib20] Zhang Y, Shen L, Yin X, et al. 2022 Live-birth prediction of natural-cycle in vitro fertilization using 57,558 linked cycle records: a machine learning perspective. Front Endocrinol 13 838087. (10.3389/fendo.2022.838087) PMC9072737 35527994

